# Supplementary material for: The influence of geostrophic strain on oceanic ageostrophic motion and surface chlorophyll
Source: Nat Commun. 2019 Jun 28;10:2838. doi: 10.1038/s41467-019-10883-w (PMC6599054; doi:10.1038/s41467-019-10883-w)
Supplement: Supplementary file 1 — Supplementary Information [file 41467_2019_10883_MOESM1_ESM.pdf]

Supplementary Information

Accompanying the article "The Influence of Geostrophic Strain on Oceanic Ageostrophic Motion and Surface Chlorophyll"

by

Zhengguang Zhang[1,2], Bo Qiu[3,*], Patrice Klein[4] & Seth Travis[3]

[1]Physical Oceanography Lab, Qingdao Collaborative Innovation Center of Marine Science and Technology, Ocean University of China, Qingdao, P. R. China.
[2]Laboratory for Ocean and Climate Dynamics, Qingdao National Laboratory for Marine Science and Technology, Qingdao, P. R. China.
[3]Department of Oceanography, University of Hawaii at Manoa, Honolulu, Hawaii, USA.
[4]Laboratoire d'Océanographie Physique et Spatiale, Brest, 29200, France.
*Correspondence and requests for materials should be addressed to B.Q. (email: bo@soest.hawaii.edu)

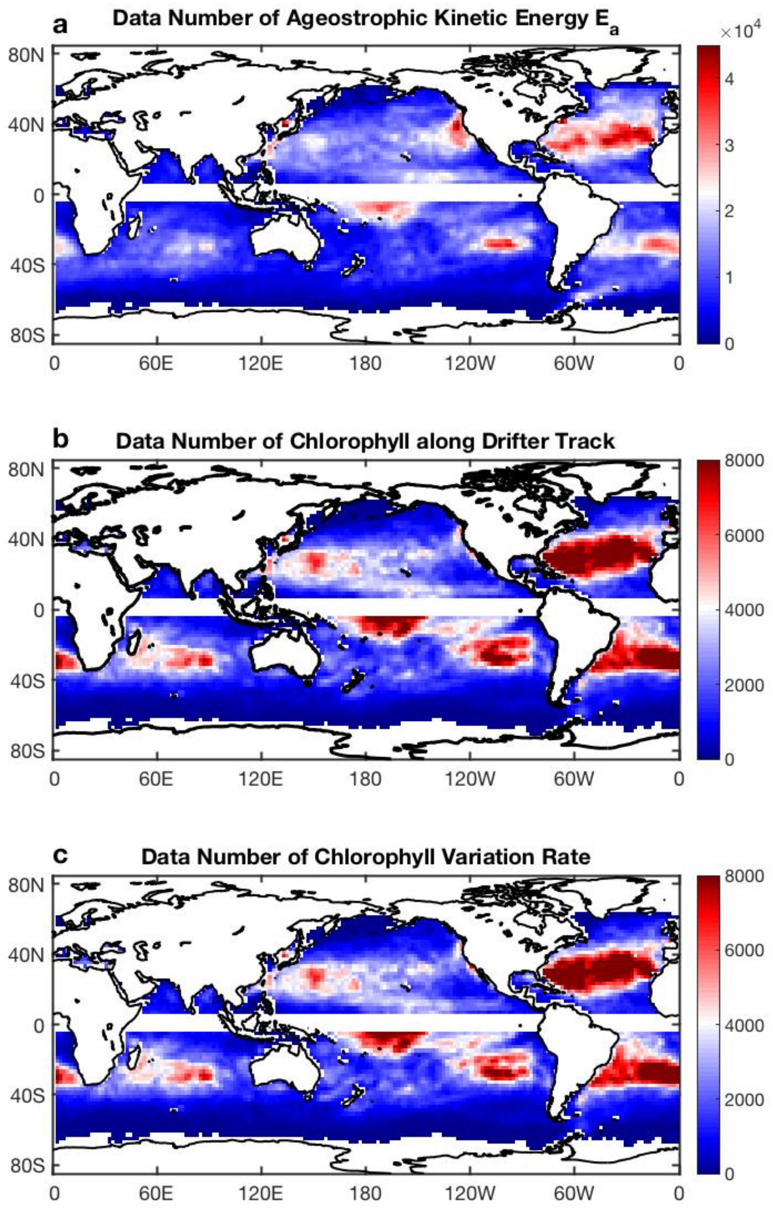

Supplementary Figure 1 | Global distribution of the number of data points within each 3°×3° average window for **(a)** ageostrophic kinetic energy **(b)** chlorophyll concentration along drifter trajectories **(c)** chlorophyll variation rate. Source data are provided as a Source Data file.

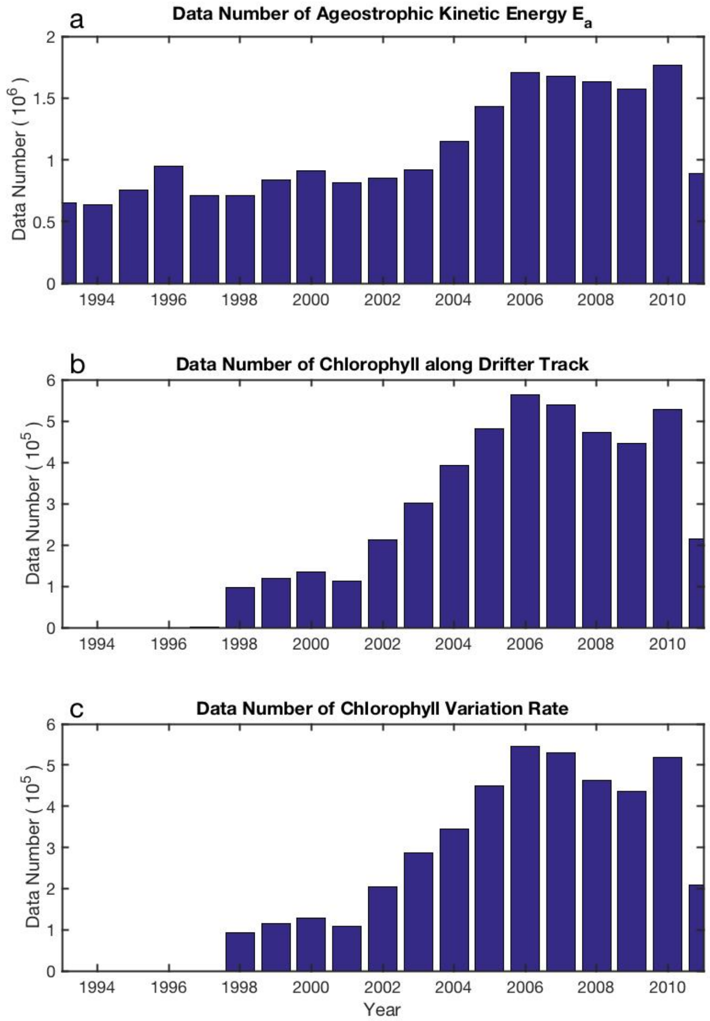

Supplementary Figure 2 | The yearly variation of the total number of data points of **(a)** ageostrophic kinetic energy **(b)** chlorophyll concentration along drifter trajectories (**c**) chlorophyll variation rate. Source data are provided as a Source Data file.

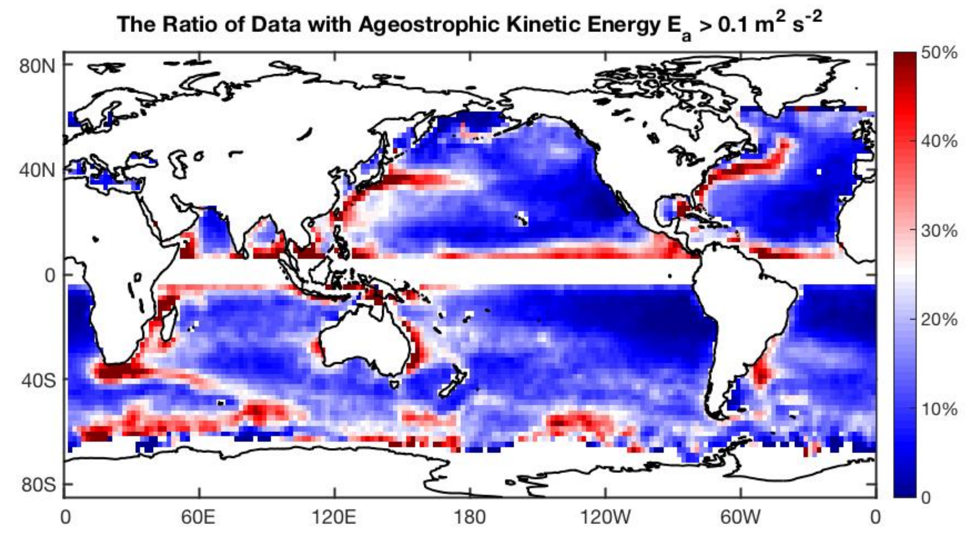

Supplementary Figure 3 | Global distribution of the ratio between the number of data points with $E_a > 0.1$ m^2 s^{-2} and the total number of data points within each 3°×3° average window. Source data are provided as a Source Data file.

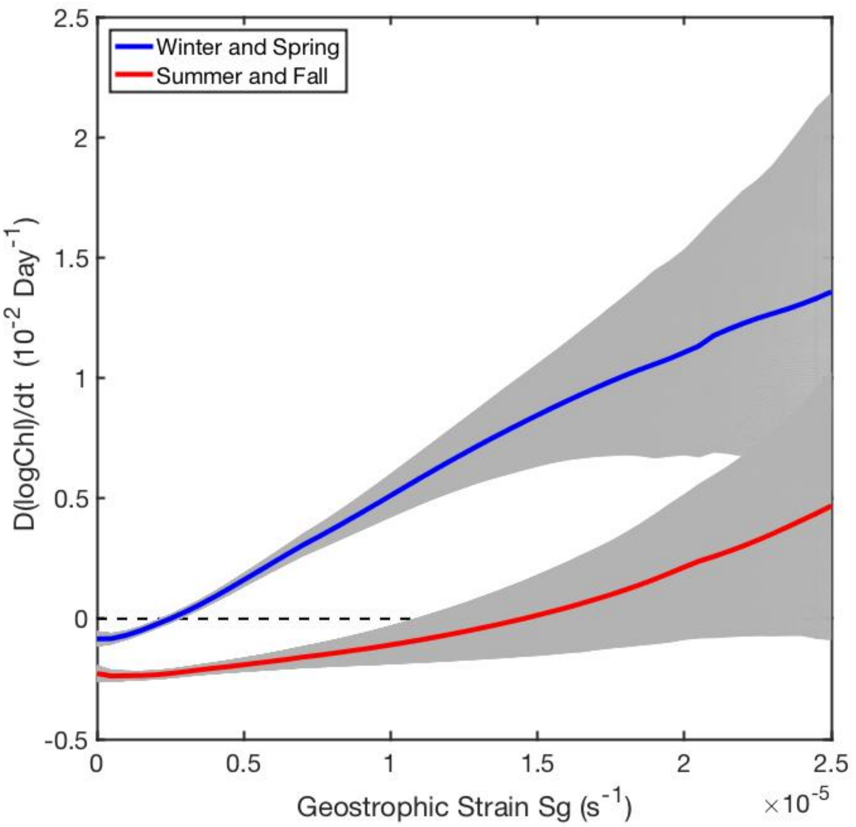

Supplementary Figure 4 | Globally-averaged curves of chlorophyll variation rate *D(logChl)/Dt* as a function of local geostrophic strain rate S_g for the winter-spring half-year (blue curve) and summer-fall half-year (red curve). These functions are computed following the same procedure as Fig.2c in the main text. Source data are provided as a Source Data file.

Supplementary Note 1| Data Coverage and Period

The global distributions of the number of data points of the ageostrophic kinetic energy E_a, the chlorophyll concentration and the chlorophyll variation rate are computed. As shown in Supplementary Figure 1, there is no general consistency between the global patterns of the distribution of the number of data points of E_a and of ageostrophic kinetic energy distribution in Supplementary Figure 1c of the main text. There is also no general consistency between the global patterns of the distribution of the number of data points of chlorophyll and its variation rate *D(logChl)/Dt* in Fig.7 of the main text. These results imply that the global patterns of ageostrophic kinetic energy and chlorophyll variation rate are not biased by the data coverage.

When computing the ageostrophic velocity and kinetic energy, only the concurrent drifter and altimetry data are used. As shown in Supplementary Figure 2a, the period for ageostrophic velocity and kinetic energy is from 1993 to 2011. When computing the chlorophyll and its variation rate, only the concurrent drifter and ocean color data are used. As shown in Supplementary Figure 2b and Supplementary Figure 2c, the period for the chlorophyll and its variation rate is from 1998 to 2011. Since the period of the ageostrophic energy data covers that of the chlorophyll data, the period is from 1998 to 2011 whenever the calculation of the chlorophyll data is involved.

In Supplementary Figure 3, the global map of the ratio between the number of data points with E_a > 0.1 m^2 s^{-2} and the total number of data points within each 3°×3°average window. It shows that a substantial part (nearly 40%-50%) of the drifter data with E_a larger than 0.1 m^2 s^{-2} is within the strong current regions. At the same time, in the western part of the subtropical gyres, about 20%-30% of the drifter data has E_a > 0.1 m^2 s^{-2}. Since chlorophyll can bloom during these strong E_a events, it is expected that submesoscale ageostrophic processes will significantly contribute to the primary production even within the subtropical gyres.

Supplementary Note 2| Seasonality of the relation between *D(logChl)/Dt* and *S*g

Both the submesoscale energy level and the phytoplankton growth condition have a strong seasonal cycle; because of this, a seasonal variation of the chlorophyll response to the strain field is expected. Since the data record is not long enough for a monthly computation of the relation between the geostrophic strain and the chlorophyll variation rate, we compute it only for the winter-spring half-year and summer-fall half-year periods, as shown in Supplementary Figure 4.

The winter-spring half-year represents November-April for the northern hemisphere (May-October for the southern hemisphere). The summer-fall half-year represents May-October for the northern hemisphere (November-April for the southern hemisphere). We find that the chlorophyll response of the winter-spring half-year is stronger than the summer-fall half-year. This is consistent with the result that the submesoscale energy level is higher during the winter-spring half-year as pointed out by recent observational studies[1-3].

Supplementary Reference

1. Callies, J., Ferrari, R., Klymak, J. M. & Gula, J. Seasonality in submesoscale turbulence. *Nat. Commun.* **6**, 6862, doi:10.1038/ncomms7862 (2015).
2. Buckingham, C. et al. Seasonality of submesoscale flows in the ocean surface boundary layer. Geophys. Res. Lett. **43**, doi:10.1002/2016GL068009 (2016).
3. Qiu, B., Nakano, T., Chen, S. & Klein, P. Submesoscale transition from geostrophic flows to internal waves in the northwestern Pacific upper ocean. *Nat. Commun*. **8**, 14055. doi: 10.1038/ncomms14055 (2017).
